# Comparisons of Jaw Line and Face Line after Mandibular Setback: Intraoral Vertical Ramus versus Sagittal Split Ramus Osteotomies

**DOI:** 10.1155/2018/1375085

**Published:** 2018-12-18

**Authors:** Chun-Ming Chen, Yu-Chuan Tseng, Edward Chengchuan Ko, Michael Yuan-Chien Chen, Kwei-Jing Chen, Jung-Hsuan Cheng

**Affiliations:** ^1^Department of Oral and Maxillofacial Surgery, Kaohsiung Medical University Hospital, Kaohsiung Medical University, Kaohsiung, Taiwan; ^2^School of Dentistry, College of Oral Medicine, Kaohsiung Medical University, Kaohsiung, Taiwan; ^3^Department of Orthodontics, Kaohsiung Medical University Hospital, Kaohsiung, Taiwan; ^4^School of Dentistry, College of Medicine, China Medical University, Taichung, Taiwan; ^5^Department of Dentistry, China Medical University Hospital, China Medical University, Taichung, Taiwan

## Abstract

**Background:**

This study investigates the differences in the lateral profile and frontal appearance after sagittal split ramus osteotomy (SSRO) and intraoral vertical ramus osteotomy (IVRO) procedures for the correction of mandibular prognathism.

**Methods:**

Sixty patients (30 SSRO and 30 IVRO) underwent mandibular setback surgery. Serial cephalograms were obtained: (1) T1: approximately 1 month before surgery; (2) T2: at least 6 months after surgery for SSRO and at least 1 year after surgery for IVRO. The landmarks, linear distances, and related angles were measured. The* t*-test was applied to the intragroup and intergroup comparisons. The null hypothesis was that SSRO and IVRO made no difference in the facial appearance.

**Results:**

In the IVRO group, the ramus and gonial widths significantly decreased by 3.9 mm and 5.8 mm, respectively. SSRO significantly reduced the gonial angle by 2.6°, and IVRO increased it significantly by 5.3°. The postoperative increases at frontal bone levels 0 and 1 after IVRO were significantly larger than those after SSRO, but, at level 3, the increases after SSRO were larger than those after IVRO. In the frontal muscular and facial planes, SSRO and IVRO presented no difference. The frontal jaw angle and face angle were significantly larger with IVRO than with SSRO. Therefore, the null hypothesis was rejected.

**Conclusions:**

The ramus width and gonial width were significantly decreased in IVRO compared to SSRO. IVRO increased angles in the lateral profile (gonial angle and mandibular plane angle) and frontal appearance (jaw angle and face angle) more than SSRO did.

## 1. Introduction

Facial aesthetics is an essential factor that determines interpersonal relationships, affects social and psychological development, and plays an important role in a person's employment and social status. Mandibular prognathism is an Angle's Class III malocclusion commonly characterized by a concave facial shape. In addition to abnormalities in the growth between the maxilla and the mandible, patients with Angle's Class III malocclusion have a shorter anterior cranial base, an acute cranial base angle, and a more obtuse gonial angle [1, 2]. Moreover, patients with mandibular prognathism present with anterior crossbite leading to difficulty in mastication. This further results in problems with malnutrition and vocalization. The unaesthetic profile and malocclusion often lead to social dysfunction and psychological disorders. However, the etiology of mandibular prognathism is still uncertain. It has been thought that environmental and genetic factors are involved in the growth and development of mandibular prognathism [1].

Treatment for patients with mandibular prognathism not only requires mandibular setback to correct the malocclusion and restore the masticatory function but also requires consideration of the harmony of facial patterns after surgery. Numerous types of mandibular setback methods exist, sagittal split ramus osteotomy (SSRO) and intraoral vertical ramus osteotomy (IVRO) being the most commonly used at present. Both SSRO and IVRO have their share of advantages and disadvantages due to the difference in osteotomy line designs. Most reports in the literature have investigated the postoperative skeletal stability [3–6] and profile changes [7–11] after these two types of surgery, but rarely have studies compared the designs of osteotomy in both procedures and their effects, especially regarding differences in bone tissue, muscular tissue, and skin surface. This study aims to investigate and compare changes between the SSRO and IVRO procedures regarding the frontal and lateral bone plane (jaw line), muscular line, and the skin surface (face line).

## 2. Materials and Methods

This study enrolled 60 patients with mandibular prognathism. The exclusion criteria were as follows: (1) patients with craniofacial malformations such as a cleft lip and palate; (2) patients who have had facial trauma or tumors; (3) patients who underwent surgeries like genioplasty or maxillary surgery. Thirty patients (17 men and 13 women), with an average age of 24 years (range of 18–33 years), underwent SSRO setback mandible and miniplate rigid fixation at the Department of Oral and Maxillofacial Surgery, China Medical University Hospital. The other 30 patients (12 men and 18 women), with an average age of 20.6 years (range of 17–34 years), underwent modified IVRO [12] and 6-week intermaxillary fixation (IMF) at the Department of Oral and Maxillofacial Surgery, Kaohsiung Medical University Hospital.

**Figure 1:**
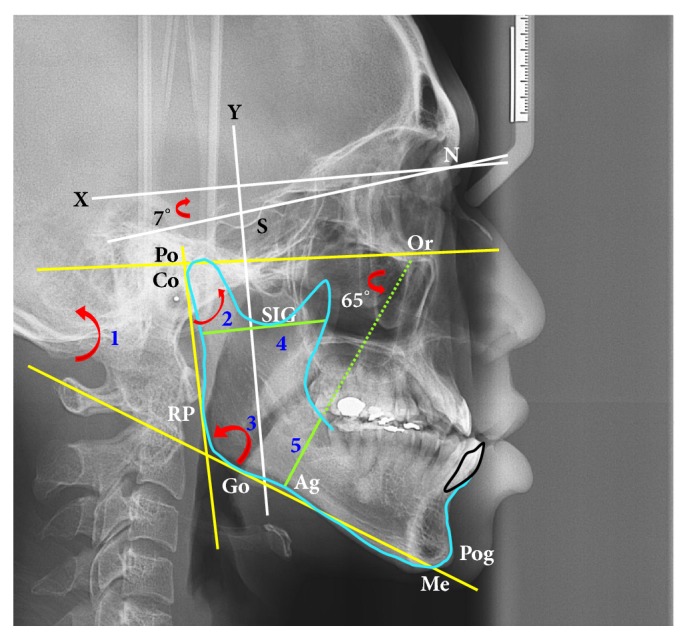
Cephalometric landmarks, linear and angular measurements. N: nasion, S: sella, Po: porion, Or: orbitale, Pog: pogonion, Me: menton, SIG: sigmoid notch, Ag: antegonial notch, Go: gonion, Co: condylion, and RP: the posteriormost and inferiormost points of the ramus.* x*-axis (horizontal line: 7° to NS line),* y*-axis (vertical line through S). FH plane: a line connecting Po to Or. Pterygomasseteric sling (PMS) plane: a line through Ag point 65° to FH plane. Red arrow (angle): 1: mandibular plane angle; 2: ramus angle; 3: gonial angle. Green lines (distances): 4: ramus width; 5: gonial width.

Serial cephalograms were obtained: (1) T1: approximately 1 month before surgery; (2) T2: at least 6 months after surgery for SSRO and at least 1 year or more after surgery for IVRO. The following landmarks were identified: sella (S), nasion (N), orbitale (Or), porion (Po), condylion (Co), the posteriormost and inferiormost points of the ramus (RP), gonion (Go), pogonion (Pog), antegonial notch (Ag), sigmoid notch (SIG), and menton (Me). This coordinate system had its origin at point N and its* x*-axis at an upward 7° of the sella-nasion (S-N) line as the horizontal axis. The* y*-axis was the line passing through the S and perpendicular to the* x*-axis (Figure 1). The ramus width (ramus distance through the SIG parallel to the FH line) and gonial width (gonial distance through the Ag point 65° to the FH line) [13] were measured (Figure 1). The three angles (ramus angle, gonial angle, and mandibular plane angle) were investigated using lateral cephalography.

**Figure 2:**
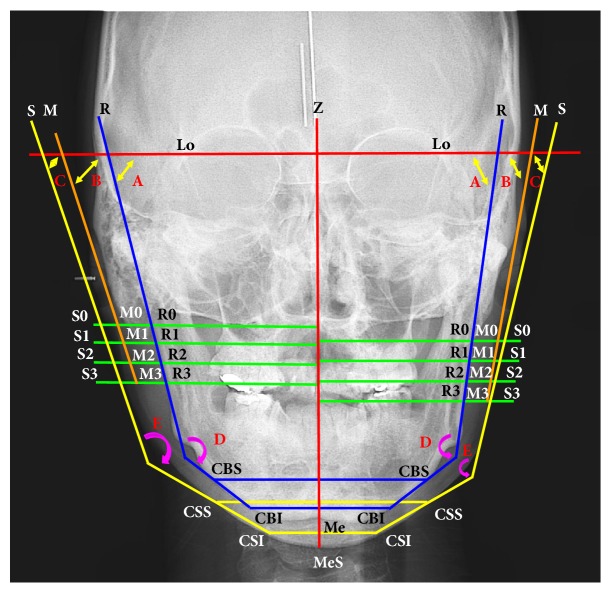
Lo: lateral orbitale, horizontal plane (Lo-Lo line),* z*-axis (red line): midsagittal line perpendicular to Lo-Lo line, Me: menton, MeS: soft tissue of Me, R line (bone plane: R0-R3 line), M line (muscular plane: M0-M3 line), and S line (skin plane: S0-S3 line). CSI: chin skin inferior, CBI: chin bone inferior, CSS: chin skin superior, and CBS: chin bone superior. Green lines (distances): R line, M line, and S line at level 0 to level 3. Yellow double arrow (angles): A: Or-ramus angle, B: Or-muscle angle, and C: Or-skin angle. Pink arrow (angles): D: jaw angle (angle between R line and CBS-CBI line), E: face angle (angle between S line and CSS-CSI line).

In the posteroanterior film, the Lo (lateral orbitale) is the intersection of the Lo contour with the innominate line. The Lo-Lo line is as the horizontal plane and the* z*-axis is a midsagittal line perpendicular to the horizontal plane. R0 (ramus origin) is the most lateral inferior point, where the mastoid process outline crosses the condylar neck. R1 is 10 mm below R0 and so on. From clinical observation, the long axis of the canine was a junction with the mandibular inferior border at the CBI (chin bone inferior; 5 mm above Me). The long axis of the second molar was a junction with the mandibular inferior border at the CBS (chin bone superior; 20 mm above Me). Therefore, the study's design was as follows: CSI (chin skin inferior) and the CBI are 5 mm above the MeS (skin of menton) and the Me (bone of menton), respectively. The CSS (chin skin superior) and the CBS (chin bone superior) are 20 mm above the MeS and Me, respectively. The R line (bone plane) is connected with R0 and R3. The M line (muscular plane) is connected with M0 and M3. The S line (skin plane) is connected with S0 and S3. The chin bone line is connected with the CBS and the CBI. The chin skin line is connected with the CSS and the CSI. The angles (A: Or-ramus angle, B: Or-muscle angle, and C: Or-skin angle) are the intersection of the Lo-Lo line with the R line, M line, and S line, respectively. The jaw angle (angle D) is the angle between the R line and CBS-CBI line. The face angle (angle E) is the angle between the S line and CSS-CSI line. In this study, bilateral frontal distances were measured in the bone, muscle, and skin planes. The postoperative changes in frontal dimensions and angles were measured (Figure 2).

The data in this study were analyzed using the IBM SPSS 20 statistical software at a statistically significant value* p* < 0.05. The* t*-test was applied to the intragroup and intergroup comparisons. The null hypothesis was that SSRO and IVRO made no difference in the facial appearance. The systematic error and accidental error from the two data collections were calculated. The systematic error was calculated using the paired* t*-test to determine whether there was a significant difference between the two descriptions and measurements. The accidental error was calculated based on the following formula (Dahlberg's formula). The Dahlberg formula is expressed as follows: accidental errors = $\sqrt{\sum d^{2}/2n}$, where d represents the difference between the two sets of data and n represents the number of measurements. Statistical analysis showed no statistically significant difference, and thus there were no systematic and accidental errors. This was a retrospective study, approved by the human investigation review committee of both hospitals.

## 3. Results

Table 1 shows the comparative outcomes of the SSRO and IVRO groups. Both groups revealed significant changes regarding the amount of setback after surgery. The setback in IVRO (11.9 mm) was more significant than that in SSRO (5 mm). In the vertical direction, the SSRO group had significantly superior movement whereas the IVRO did not. In the SSRO group, ramus width decreased by 0.6 mm and gonial width by 0.9 mm, and these changes were not statistically significant. In the IVRO group, ramus width and gonial width significantly decreased by 3.9 mm and 5.8 mm, respectively. In addition, the IVRO group values (ramus width and gonial width) decreased significantly more than did the SSRO group values.

**Figure 3:**
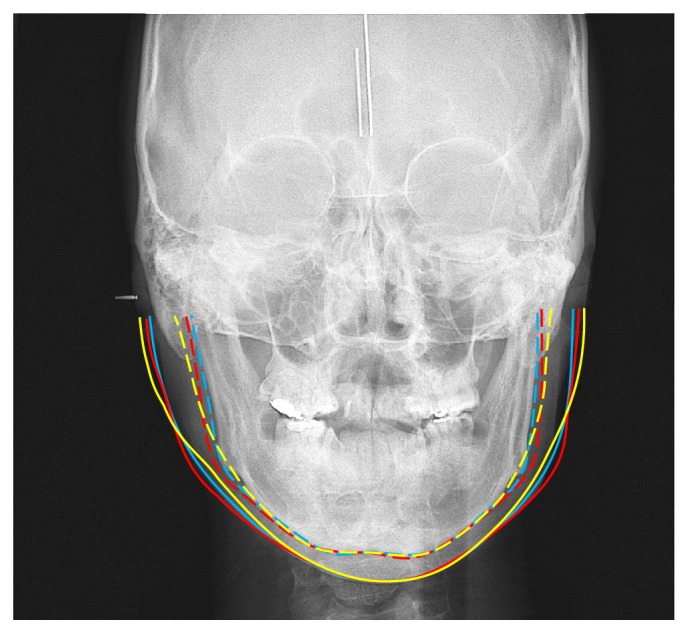
Jaw line (dotted line): preoperation (blue); postoperation (red: SSRO); postoperation (yellow: IVRO). Face line (solid line): preoperation (blue); postoperation (red: SSRO); postoperation (yellow: IVRO).

The postoperation mandibular patterns of the SSRO and IVRO groups were shown in Figure 3. Investigation of the changes in related angles revealed that SSRO significantly increased the ramus angle by 2.2° and IVRO had an insignificant increase of 0.9°. SSRO significantly reduced the gonial angle by 2.6° and IVRO significantly increased it by 5.3°. The change in the mandibular plane angle with SSRO was minimal, whereas IVRO significantly increased it by 6.2°. The increases in both the gonial and the mandibular angles were significantly larger with IVRO than with SSRO.

Table 2 reveals significantly increased postoperative frontal distance at levels (levels 0, 1, and 2) of the bone plane after both SSRO and IVRO. At bone level 3, SSRO significantly increased the frontal distance by 3.2 mm, whereas IVRO decreased it by 0.9 mm, but the difference was not significant. On intergroup comparisons, the postoperative increases at bone levels 0 and 1 were significantly larger after IVRO than after SSRO, but, at level 3, the increases after SSRO were larger than after IVRO. At levels (levels 0, 1, 2, and 3) of the muscular plane, no significant difference existed in the postoperative frontal distance after both SSRO and IVRO, and no difference existed for intergroup comparisons either. However, SSRO increased the frontal distance by 1.2 mm at muscular level 3, whereas IVRO decreased it by 0.5 mm. At levels (levels 0, 1, 2, and 3) of the facial plane, no significant difference existed in the postoperative frontal distance after both SSRO and IVRO, and no difference existed for intergroup comparisons either. However, SSRO increased the frontal distance by 1.3 mm at facial level 3, whereas IVRO decreased it by 1.4 mm.

The postoperation frontal appearances of the SSRO and IVRO groups were shown in Figure 3.  Table 3 reveals that SSRO significantly increased the frontal Or-ramus angle by 2.3° and significantly decreased the jaw angle by 3.8°. IVRO significantly decreased the Or-ramus angle by 7.3° and face angle by 5.4°. On intergroup comparisons, the amount of decrease in the Or-ramus angle and face angle was greater with IVRO than with SSRO. IVRO significantly increased the jaw angle and face angle by 7.1° and 3.4°, respectively, whereas SSRO decreased the jaw angle and face angle by 3.8° and 3.5°, respectively. Intergroup comparisons showed that the increases in jaw angle and face angle were larger with IVRO than with SSRO. Therefore, the null hypothesis was rejected.

## 4. Discussion

Both SSRO and IVRO have advantages and disadvantages due to the use of different osteotomy line designs. Generally, IVRO has a significantly lower probability of injuring the inferior alveolar nerve than does SSRO [14, 15], but IVRO requires IMF for 6 weeks, which allows the proximal and distal segments to maintain stability and undergo bone healing. Unlike IVRO, SSRO uses rigid internal fixation to bind the distal and proximal segments and does not need IMF. In the meta-analysis, Al-Moraissi and Ellis [16] found that there was no statistically significant difference in skeletal stability between bicortical screw fixation and plate fixation of the SSRO when used for mandibular setback. After SSRO, the patients can open their mouths facilitating oral intake and experience minimal hindrance in social interaction. The main disadvantages after IVRO are that patients can only consume liquid food and challenges in oral hygiene maintenance. Ideally, the surgeon and the patient should discuss the degree of injury to the inferior alveolar nerve and the effects of each method on oral intake and social interaction before proceeding with either of the treatment options.

The major differences between the osteotomy design of SSRO and IVRO are as follows. (1) In SSRO, the ramus is split into two halves and the osteotomy line extends to the mandibular molar area, but the external appearance of the ramus is unchanged. In IVRO, the ramus is cut through behind the mandibular foramen from the sigmoid notch to the mandibular angle, and the external appearance of both the ramus and the mandibular angle is changed. (2) The patterns of bone overlap are different. The two halves are reunited in SSRO, whereas, in IVRO, the two intact segments are overlapped and its thickness doubled resulting in a changed frontal appearance. Investigating the computed tomography, Rokutanda et al. [6] reported that osseous healing was similar in patients undergoing SSRO and IVRO at the postoperative one year.

The present study reveals that the morphological change in lateral ramus width and gonial width is small and insignificant after SSRO. In IVRO, the characteristics of bone overlap are apparent as the lateral ramus and gonial widths are significantly decreased, and the external appearance of the ramus significantly changed. Therefore, the changes in external appearance associated with the changes in the lateral ramus and angle produced by IVRO are more obvious than changes produced by SSRO. As the amount of setback in IVRO increases, the amount of bone overlap and the decrease in ramus width both increase. In addition, modified IVRO excises the inferior portion of the proximal segment preventing the proximal segment from protruding beyond the mandibular inferior border and preventing the patient from having a sensation of protrusion when touching. The present study reveals that the ramus angle is still significantly increased, although Pog is setback by counterclockwise rotation in SSRO. The reasons are that the proximal segment moves clockwise backward after distal segment setback or the condyle of the proximal segment must be pushed into the glenoid fossa posteriorly and superiorly during rigid fixation of both segments, producing a significant increase in the ramus angle.

There is still controversy that the gonial angle was increased or decreased after mandibular rami osteotomies. Jönsson et al [17] reported that the gonial angle was found to increase 5° in SSRO and to decrease 3.3° in oblique sliding osteotomy. Kitahara et al. [8] reported inverse results in which the gonial angle was found to decrease by 4.4° in SSRO and to increase by 3.3° in IVRO. Despite the significant decrease in the gonial angle in SSRO in our study, the ramus angle and gonial angle exhibit a complementary relationship, and hence the small change in the mandibular plane angle is reasonable. However, IVRO has no fixation of the proximal and distal segments, so the ramus angle returns to the new physiological position based on its functional needs and the action of bone remodeling. This position corresponds to bone movement and muscle attachment, and thus the postoperative ramus angle does not change significantly, which is entirely different from what occurs with SSRO. Similarly, the gonial angle is significantly increased following postoperative remodeling in IVRO, which is unlike what takes place with SSRO. In addition, the gonial angle is significantly larger following IVRO than SSRO. Similarly, the mandibular plane angle is significantly increased following IVRO, and not with SSRO. Additionally, the mandibular plane angle is significantly larger following IVRO than that following SSRO. Regarding the external appearance of the mandibular angle area, the increases in the gonial angle and mandibular plane angle result in a smoother profile of patients.

Yoshioka et al. [18] reported that intergonial width increased by 1.21 mm in the IVRO group and 0.45 mm in the SSRO group. However, Yeo et al. [19] showed that intergonial width decreased 2.59 mm after mandibular setback by SSRO. Choi et al. [20] investigated long-term changes in mandibular and facial widths after mandibular setback surgery using IVRO. They found that frontal mandibular width increased after IVRO but seemed to normalize within approximately 3 years. In our study, the bone width at levels 0 and 1 was significantly increased in IVRO compared to SSRO and the bone width at level 3 was significantly increased in SSRO compared to IVRO. Therefore, the Or-ramus angle significantly increased by 2.3° in SSRO but significantly decreased by 7.3° in IVRO. These result in a significantly larger Or-ramus angle with SSRO than with IVRO.

Upon investigating the frontal muscle plane, no significant change existed for any of the four levels with SSRO or IVRO. Although the width in the bone plane was significantly increased with IVRO and SSRO, the muscle plane integrated the increased thickness in the bone resulting in insignificant increases in the muscle plane with IVRO and SSRO. The Or-muscle angle in SSRO was observed to be similar to the increase in its Or-ramus angle. Comparatively, in IVRO, the muscle plane integrated the change in bone thickness resulting in significantly smaller change in the Or-muscle angle than the Or-ramus angle.

Choi et al. [20] reported that frontal facial width did not reflect underlying skeletal changes after IVRO. Upon evaluating the frontal facial plane, no significant change existed for any of the four levels with SSRO or IVRO, which signifies that there was no significant increase in frontal facial width following significant frontal bone changes in SSRO and IVRO. It was observed that the increases in the level 3 bone, muscle, and facial plane widths were similar in SSRO, whereas the decreases in the level 3 bone, muscle, and facial plane widths were similar in IVRO. Our findings were similar to the report of Choi et al. [20]. The change in the Or-face angle in SSRO was small, whereas it was significantly decreased in IVRO. Therefore, the Or-face angle with SSRO was significantly larger than with IVRO.

Regarding the jaw angle, the significant increase in the Or-ramus angle in SSRO led to a significant decrease in the jaw angle. However, the significant decrease in the Or-ramus angle in IVRO led to a significant increase in the jaw angle. Hence, the jaw angle was significantly larger in IVRO than in SSRO. In IVRO, there was considerable increase in the face angle because of the significant decrease in the Or-face angle. In SSRO, there was small increase in the Or-face angle, and hence the face angle decreased correspondingly but not significantly. Therefore, the face angle was significantly larger with IVRO than with SSRO. In other words, both the jaw angle and face angle were significantly larger with IVRO than with SSRO.

In conclusion, ramus width and gonial width were significantly decreased in IVRO than SSRO. The frontal muscular and skin surface planes presented no significant difference between IVRO and SSRO. IVRO increased angles in the lateral profile (gonial angle and mandibular plane angle) and frontal profile (jaw angle and face angle) relative to SSRO.

## Figures and Tables

**Table 1:** Comparison of lateral dimensions after mandibular setback operations (SSRO vs. IVRO).

	Postoperation changes									
	SSRO				IVRO				SSRO vs. IVRO	
	Mean	SD	*p *value		Mean	SD	*p* value		*p *value	
Pog (horizontal)	-5.0	3.41	< 0.0001	*∗*	-11.9	4.15	< 0.0001	*∗*	< 0.0001	†, IVRO > SSRO
Pog (vertical)	-1.4	2.73	0.008	*∗*	-0.2	2.68	0.7355	—	0.0774	—
Ramus width	-0.6	2.16	0.1252	—	-3.9	3.05	< 0.0001	*∗*	< 0.0001	†, IVRO > SSRO
Gonial width	-0.9	3.65	0.2198	—	-5.8	2.66	< 0.0001	*∗*	< 0.0001	†, IVRO > SSRO
Ramus angle	2.2	3.03	0.0006	*∗*	0.9	4.97	0.3288	—	0.3011	—
Gonial angle	-2.6	3.77	0.0010	*∗*	5.3	4.45	< 0.0001	*∗*	< 0.0001	†, IVRO > SSRO
Mandibular plane angle	-0.4	3.42	0.5173	—	6.2	4.39	< 0.0001	*∗*	< 0.0001	†, IVRO > SSRO

*∗*: intragroup comparison: statistically significant, *p *< 0.05.

†: intergroup comparison: statistically significant, * p *< 0.05.

—: not significant.

**Table 2:** Comparison of frontal distances after mandibular setback operations (SSROvs. IVRO).

		Postoperation changes									
		SSRO				IVRO				SSRO vs.IVRO	
Bilateral sides		Mean	SD	*p *value		Mean	SD	*p* value		*p *value	
Bone plane	0	1.9	3.07	0.0030	*∗*	4.4	3.51	< 0.0001	*∗*	0.0040	†, IVRO > SSRO
	1	1.9	2.91	0.0012	*∗*	4.7	4.14	< 0.0001	*∗*	0.0044	†, IVRO > SSRO
	2	2.7	2.79	< 0.0001	*∗*	3.3	4.91	0.0010	*∗*	0.5938	—
	3	3.2	4.07	0.0002	*∗*	-0.9	7.37	0.5005	—	0.0207	†, SSRO > IVRO

Muscle plane	0	0.1	4.29	0.8846	—	0.9	3.84	0.2341	—	0.6025	—
	1	0.9	3.90	0.2505	—	0.7	4.10	0.3548	—	0.7963	—
	2	0.9	4.24	0.2606	—	0.0	4.03	0.9648	—	0.3837	—
	3	1.2	5.75	0.2578	—	-0.5	6.19	0.6877	—	0.2604	—

Skin plane	0	0.5	4.14	0.5345	—	1.2	5.18	0.2284	—	0.6785	—
	1	-0.5	4.09	0.5580	—	0.3	5.61	0.7874	—	0.6460	—
	2	0.2	5.18	0.8635	—	-0.9	5.75	0.4063	—	0.4108	—
	3	1.3	5.00	0.1720	—	-1.4	6.70	0.2751	—	0.0995	—

*∗*: intragroup comparison: statistically significant, *p *< 0.05.

†: intergroup comparison: statistically significant, * p *< 0.05.

—: not significant.

**Table 3:** Comparison of frontal angles after mandibular setback operations (SSROvs. IVRO).

	Postoperation changes									
	SSRO				IVRO				SSRO vs.IVRO	
Bilateral sides	Mean	SD	*p *value		Mean	SD	*p* value		*p *value	
Or-ramus angle	2.3	5.53	0.0326	*∗*	-7.3	11.15	0.0015	*∗*	0.0001	†, SSRO > IVRO
Or-muscle angle	2.9	11.36	0.1749	—	-0.5	9.56	0.7874	—	0.1917	—
Or-skin angle	0.7	7.50	0.5965	—	-5.4	6.51	0.0001	*∗*	0.0010	†, SSRO > IVRO
Jaw angle	-3.8	6.95	0.0052	*∗*	7.1	9.99	0.0007	*∗*	0.0001	†, IVRO > SSRO
Face angle	-3.5	11.48	0.1073	—	3.4	7.66	0.0229	*∗*	0.0054	†, IVRO > SSRO

*∗*: intragroup comparison: statistically significant, *p *< 0.05.

†: intergroup comparison: statistically significant, * p *< 0.05.

—: not significant.

## Data Availability

The data used to support the findings of this study are available from the corresponding author upon request.
